# Investigation of Pathogenic Mechanism of Covert Mortality Nodavirus Infection in *Penaeus vannamei*

**DOI:** 10.3389/fmicb.2022.904358

**Published:** 2022-05-31

**Authors:** Shuang Liu, Jitao Xia, Yuan Tian, Liang Yao, Tingting Xu, Xupeng Li, Xiaoping Li, Wei Wang, Jie Kong, Qingli Zhang

**Affiliations:** ^1^Yellow Sea Fisheries Research Institute, Chinese Academy of Fishery Sciences, Qingdao, China; ^2^Key Laboratory of Maricultural Organism Disease Control, Ministry of Agriculture and Rural Affairs, Qingdao, China; ^3^Qingdao Key Laboratory of Mariculture Epidemiology and Biosecurity, Qingdao, China; ^4^Laboratory for Marine Fisheries Science and Food Production Processes, National Laboratory for Marine Science and Technology, Qingdao, China

**Keywords:** pathogenic mechanism, viral covert mortality disease (VCMD), running mortality syndrome (RMS), covert mortality nodavirus (CMNV), slow growth, neurotropism

## Abstract

Viral covert mortality disease (VCMD), also known as running mortality syndrome (RMS), is caused by covert mortality nodavirus (CMNV) and has impacted the shrimp farming industry in Asia and Latin America in recent years. The pathogenic mechanism of CMNV infecting *Penaeus vannamei* was investigated in this study. In the naturally infected shrimp, histopathological and *in situ* hybridization (ISH) analysis verified that CMNV infection and severe cellar structural damage occurred in almost all cells of the ommatidium. Under transmission electron microscopic (TEM), vacuolation and necrosis, together with numerous CMNV-like particles, could be observed in the cytoplasm of most cell types of the ommatidium. The challenge test showed that a low CMNV infectious dose caused cumulative mortality of 66.7 ± 6.7% and 33.3 ± 3.6% of shrimp in the 31-day outdoor and indoor farming trials, respectively. The shrimp in the infection group grew slower than those in the control group; the percentage of soft-shell individuals in the infection group (42.9%) was much higher than that of the control group (17.1%). The histopathological and ISH examinations of individuals artificially infected with CMNV revealed that severe cellar damage, including vacuolation, karyopyknosis, and structural failure, occurred not only in the cells of the refraction part of the ommatidium, but also in the cells of the nerve enrichment and hormone secretion zones. And the pathological damages were severe in the nerve cells of both the ventral nerve cord and segmental nerve of the pleopods. TEM examination revealed the ultrastructural pathological changes and vast amounts of CMNV-like particles in the above-mentioned tissues. The differential transcriptome analysis showed that the CMNV infection resulted in the significant down-regulated expression of genes of photo-transduction, digestion, absorption, and growth hormones, which might be the reason for the slow growth of shrimp infected by CMNV. This study uncovered unique characteristics of neurotropism of CMNV for the first time and explored the pathogenesis of slow growth and shell softening of *P. vannamei* caused by CMNV infection.

## Introduction

Aquaculture, including shrimp farming, remains the world’s fastest-growing sector producing food of animal origin (Kibenge, 2019). In the past decade, emerging aquaculture viruses have significantly impacted world aquaculture and threatened world food security (Tandel et al., 2017; Megahed et al., 2018; Kibenge, 2019; FAO, 2020). Among the viruses reported in shrimp, covert mortality nodavirus (CMNV) is a newly found virus isolated from white leg shrimp *Penaeus vannamei* (Zhang et al., 2014, 2017). CMNV was identified to be the pathogenic agent of shrimp viral covert mortality disease (VCMD), which has caused serious production losses in the shrimp farming industry (Zhang et al., 2014, 2017, 2019; Pooljun et al., 2016). CMNV is prevalent in China and countries in Southeast Asia, and Latin America (Varela, 2018; Zhang et al., 2018; Kibenge, 2019).

In outdoor ponds, VCMD causes low mortality in the affected shrimp every day. This daily continuous mortality occurs throughout the culture period; so VCMD was initially called “running mortality syndrome (RMS)” (Zhang et al., 2017; Zhang, 2019). Higher mortalities occur at water temperatures above 28°C and with sudden changes in weather (Zhang et al., 2014, 2017). In indoor tank farming, VCMD usually does not cause obvious mortality in the affected shrimp unless there are sudden changes in environmental conditions.

The virus has a wide host range among invertebrates, and it is known to infect the major cultured shrimp species, as well as the co-inhabiting organisms in shrimp ponds (Zhang et al., 2017; Liu et al., 2018; Li et al., 2019). Recently, natural infections of CMNV were reported in several species of fish such as *Mugilogobius abei*, a common marine fish in shrimp ponds and coastal water in China, and marine fish *Chaeturichthys hexanema* and *Larimichthys polyactis* from the Yellow Sea, and farmed Japanese flounder *Paralichthys olivaceus*. Thus, this virus is capable of naturally crossing the species barrier and infecting both vertebrates and invertebrates (Zhang et al., 2018; Wang et al., 2019a,b; Wang et al., 2021; Xu et al., 2021).

Although CMNV has become a threat to farmed shrimp in major aquaculture countries, the mechanism by which it infects shrimp and causes the disease is still unclear, Epidemiological investigations and research in the authors’ laboratory found that natural CMNV infection causes retina lesions in the fish, and this pathogenic feature indicates a neurotropic effect of CMNV similar to that of *Betanodavirus*.

The lack of pathogenesis knowledge of CMNV largely limits shrimp farming practitioners from implementing effective prevention and control measures against VCMD. Therefore, this study attempted to explore the pathogenesis of CMNV infection in shrimp by investigating naturally infected and artificially infected *P. vannamei* individuals through the use of molecular histopathological analysis, *in situ* hybridization, transmission electron microscopic (TEM), and differential transcriptome assays.

## Materials and Methods

### Shrimp Sample

Samples of farming *P. vannamei* (body length 5–7.5 cm) were collected from earth ponds suffering an outbreak of VCMD on a farm in Weifang city in Shandong Province in November 2016 in our epidemiological investigation. These shrimp samples were tested to be free of white spot syndrome virus (WSSV), infectious hypodermal and hematopoietic necrosis virus (IHHNV), shrimp hemolytic iridescent virus (SHIV), acute hepatopancreatic necrosis disease caused by *Vibrio parahaemolyticus* (*Vp*_AHPND_), enterocytozoon hepatopenaei (EHP), yellow head virus (YHV) and hepatopancreatic parvovirus (HPV). Whereas, the samples were CMNV positive in the CMNV reverse transcription nested PCR (RT-nPCR) test. And the CMNV RT-nPCR amplicons were then sent for sequencing to the commercial sequencing company of Shanghai SANGAN Chemical Trading Co. Ltd.

For the challenge test, specific-pathogen-free (SPF) juvenile *P. vannamei* shrimps were collected from Haixingnong Shrimp Breeding Northern Base of BLUMP Seed Industry Technology Co., Ltd. in Weifang city of Shandong Province. The juvenile shrimps were tested to be free of WSSV, IHHNV, *Vp*_AHPND_, EHP, YHV, and HPV in the PCR or RT-PCR assays recommended by the OIE Aquatic Manual (OIE, 2019) and previous reports (Liu et al., 2013; Tang et al., 2015).

### Phylogenetic Analysis

The gene fragment of CMNV RNA-dependent RNA polymerase (RdRp) (413 nt from nt no. 357 to 769 in the reference sequence of GenBank accession number KM112247) cloned from samples of the VCMD case in the present study was first translated into amino acid sequence and then used for the phylogenetic analysis. Both the deduced CMNV RdRp amino acid sequence, and the relevant homologous sequences in the family Nodaviridae retrieved from the GenBank database, were submitted for aligning analysis by using the ClustalW multiple alignment algorithm in the MEGA 3.1 according to the previous reports (Hall, 2013).

### Histopathological Section

Samples of the shrimp eyestalks, cephalothoraxes, abdominal segment, and swimming legs were fixed in 4% PFA fixative for 24 h and then fixed in 70% ethanol. Paraffin sections were prepared and stained with H&E staining according to the procedures reported by Bell and Lightner (1988). Triplicate paraffin sections (3 μm) were prepared for histological and ISH analysis. The first sections of every sample were stained with routine H&E-phloxine according to Lightner’s protocols (Lightner, 1996). After checking the H&E-stained sections, the left sections of every sample were subjected to CMNV ISH assay with digoxigenin (DIG)-labeled RNA probe.

### *In situ* RNA Hybridization

The CMNV RNA probe used for ISH was synthesized according to the previous report (Zhang et al., 2018). The ISH of each sample was conducted referring to the protocols described in a previous study (Chen et al., 2014). After counterstained with Nuclear Fast Red following the method reported before (Nuovo et al., 1999), the sections were mounted with water-soluble sealant for further examination by Nikon Eclipse E80i microscope (Nikon Co., Tokyo, Japan).

### Transmission Electron Microscopy

The sample tissue in < 1 mm^3^ was fixed in TEM fixative (2% paraformaldehyde, 2.5% glutaraldehyde, 160 mM NaCl, and 4 mM CaCl_2_ in 200 mM PBS) (pH 7.2) for 24 h at 4°C and was subjected to further fixation with 1% osmium tetroxide, and dehydrated in a graded ethanol series, then embedded in Spurr’s resin and prepared ultrathin sections of 50 nm in thickness. The sections were stained with uranyl acetate and lead citrate in accordance with the previously reported protocols (Graham and Orenstein, 2007). Ultrathin sections were laid on collodion-coated grids and examined by using a JEOL JEM-1200 electron microscope (Nikon Co., Tokyo, Japan).

### Virus Purification

Cephalothoraxes were dissected aseptically and homogenized in sterile PBS using a mortar and pestle. The homogenate liquid was centrifuged at 10,000 *g* for 25 min at 4°C to remove tissue debris, and the supernatant was filtered through a 22 mm membrane and then centrifuged at 130,000 *g* for 4 h. The precipitation was re-suspended as viral inoculum for the challenge test.

### Challenge Test

Healthy juvenile *P. vannamei* (about 4.5–5.7 cm in body length) were cultured temporarily in tanks for 3 days before they were used for challenge tests. For the challenge experiment, the shrimp with similar body lengths were selected first and then were divided into an infection group and a control group. It had been observed previously in shrimp aquaculture practice that after indoor cultured *P. vannamei* was infected with CMNV, the mortality rate of infected individuals would be lower than that of outdoor farming infected individuals, and the growth retarding of infected individuals in the indoor aquaculture model would also be alleviated to a certain extent. Therefore, for the convenience of comparison, this study also carried out a CMNV challenge test in indoor cultured *P. vannamei*.

For the outdoor farming challenge test, each group included two replicates and each replicate included 15 individuals due to the limited space conditions of the farming facility. For the indoor farming challenge test, each group included three replicates and each replicate included 35 individuals. Then, 5 μL of viral inoculum (5.5 × 10^6⋅5^ copies/μL, about 2 × 10^4⋅5^ viral copies per mg shrimp) (or TN buffer) were injected intramuscularly into the third abdominal segment of each healthy shrimp individual to be serving as the infection group (or the control group). Thereafter, the shrimp were maintained with pellet feeds for 31 days in tanks locating outdoor and indoor environments and the mortality were recorded daily. In the outdoor challenge test, the water temperature in the culture tanks varied from 18 to 33°C with the change in outdoor temperature. In the indoor challenge test, the water temperature in the culture tanks was maintained at 30–32°C. The salinity of the seawater used is 29–30 parts per thousand. During the challenging trial, the daily water renewal is about one-third of each aquaculture tank. At the end of the bioassay, the body lengths of shrimp individuals were measured. The eyestalks, cephalothoraxes, abdominal segment, and swimming legs of every living shrimp sample were collected and preserved in RNAlater solution (Qiagen GmbH, Hilden, Germany), Davidson’s alcohol formalin acetic acid (AFA) solution, and 2.5% glutaraldehyde solution, respectively, for further analysis of molecular detection, histopathology and TEM analysis.

### Determination of Calcium Element in Carapace

In order to determine the calcium content in the carapace of the cephalothoraxes of the diseased and healthy shrimps, the carapace of the same location in cephalothoraxes of the diseased and healthy shrimps was fixed in TEM fixative firstly, subsequently subjected to further fixation with 1% osmium tetroxide, then dehydrated, and embedded in Spurr’s resin and prepared ultrathin sections of 50 nm in thickness. The sections were coated with a thin layer of gold-palladium alloy following the previously reported protocols (Letchumanan et al., 2020; Wagner et al., 2020), and then examined by using X-Max80 EDS (Oxford Instruments, Oxford, Britain) Field Emission Scanning Electron Microscopy (FESEM) at 20 KV. The specimen embedded in Spurr’s resin was also coated with a thin layer of gold-palladium and then inspected by using the FESEM to conduct energy-dispersive X-ray spectroscopy (EDS) to obtain the data of elemental weight percent (%) in dissociation of the main constituent elements in cephalothorax carapace.

### Differential Expression Analysis

In order to reveal the possible molecular mechanism of the slow growth of *P. vannamei* caused by CMNV infection, differential transcriptome analysis was conducted by using the total RNA of cephalothoraxes both from the healthy and infected shrimp individuals. Three total RNA samples from the healthy shrimp individuals and three total RNA samples from the CMNV infected shrimp individuals were prepared and used for the differential transcriptome analysis. Firstly, mRNA was enriched by Oligo(dT) beads from the total RNA samples, and then mRNA was fragmented and reverse transcription into cDNA. Secondly, the cDNA fragments were purified, end-repaired, poly(A) added, and ligated to Illumina sequencing adapters according to previous reports (de Wit et al., 2014; Rajagopala et al., 2018). Thirdly, the ligation products were PCR amplified and sequenced using Illumina HiSeq TM 2500. Low-quality reads, adapter sequences, as well as rRNA, were removed from the raw reads obtained from sequencing machines. The clean reads were used for transcripts reconstruction by using the software Cufflinks (Trapnell et al., 2012), together with TopHat2. Novel gene transcripts identification and annotation, quantification of gene abundance, relationship analysis of samples, and differentially expressed genes analysis were carried out based on the protocols of Gene *Denovo* Biotechnology Co. (Guangzhou, China).

### Experimental Validation of Differentially Expressed Genes

Four differentially expressed genes (DEGs) in top KEGG enrichment were selected for the qRT-PCR analysis for their expression validation in the CMNV naturally infected samples. 18S rRNA was used as an internal control. Gene-specific primers were designed using PrimerQuest^®^ Tool^1^. All primer sequences were listed in Supplementary Table 1. One-Step TB Green PrimeScript RT-PCR Kit II (TaKaRa, Dalian, China) was used for obtaining amplification curves following the manufacturer’s protocol. The melting curve analysis was conducted at the end of each PCR reaction to confirm that the specific PCR product was amplified and detected. The expression of each gene was quantified relative to that of 18S rRNA with the 2^–ΔΔCt^ comparative *Ct* method. Significance was defined as *P* < 0.05.

## Results

### Phylogenetic Analysis of the Covert Mortality Nodavirus Isolated From Typical Viral Covert Mortality Disease Case

Phylogenetic analysis based on the partial CMNV_RdRp amino acid sequence from positive shrimp samples indicated that the new identified CMNV (CMNV_Shandong) isolate from shrimp individuals of the VCMD affected pond was closely clustered in one branch with the original CMNV isolate, and were obviously different from other virus members in *Alphanodavirus* (Supplementary Figure 1). The result showed that the newly identified CMNV (CMNV_Shandong) shared a close genetic relationship with the original CMNV isolates.

### Microstructural and Ultrastructural Changes Caused by Covert Mortality Nodavirus Natural Infection

Following the clue that CMNV natural infection could cause obvious retina lesions in the fish, the microstructural and ultrastructural changes of the compound eye of the shrimp individuals who suffered VCMD naturally were analyzed by using histopathological, ISH, and TEM assays.

**FIGURE 1:**
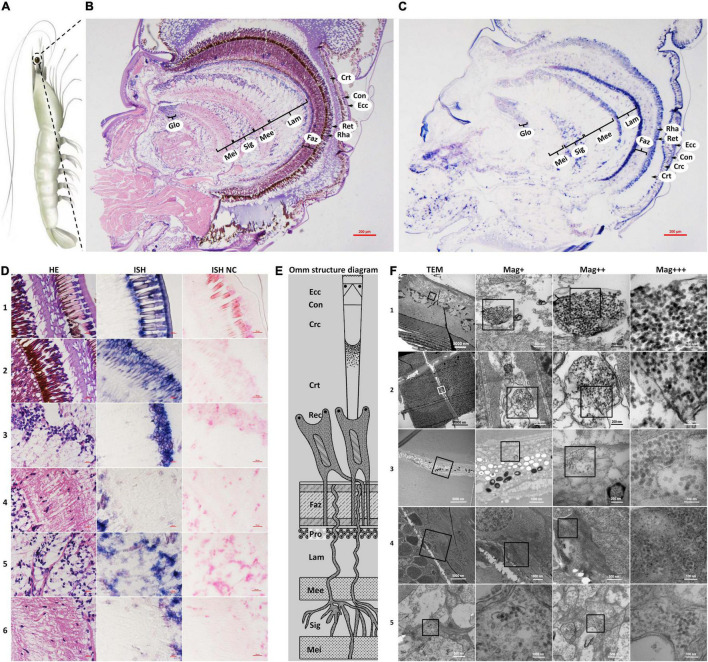
Schematic diagram of *Penaeus vannamei* and its ommatidia, and micrographs of H&E staining (HE), *in situ* hybridization (ISH), and transmission electron microscopic (TEM) of eyestalk from shrimp naturally infected with covert mortality nodavirus (CMNV). **(A)** Schematic diagram of *P. vannamei*. **(B)** Micrographs of H&E staining. **(C)** Micrographs of ISH. **(D)** Enlarged areas of micrographs of H&E staining and ISH in **(B,C)**. It can be noted that vacuolation, karyopyknosis, and structural failure in almost all types of cells that make up the ommatidium, as well as the nerve tissue of eyestalk. Note the purple hybridization signal at the cells of the ommatidium and the nerve tissue of eyestalk which demonstrated apparent vacuolation. **(D1)** Showed the Ecc, Con, Crc, and Crt; **(D2)** showed the Crt, Rec, rhabdom (Rha), and Faz; **(D3)** showed the cell rind nuclei (Crn) of the lamina ganglionaris and Lam; **(D4)** showed the Mee; **(D5)** showed the Sig; **(D6)** showed the Mei. **(E)** Schematic diagram of ommatidia structure of shrimp *P. vannamei*. Ecc, epicorneagenous cells cytoplasm; Con, cone cells; Crc, crystalline cones; Crt, crystalline tracts; Rec, retinular cell (including the Rha and Ret); Faz, fasciculated zone; Pro, primary optic nerve fibers; Lam, lamina ganglionaris; Mee, medulla externa; Sig, sinus gland; Mei, medulla interna. The same Schematic diagram has been included in Figures 1, 3, 4 for convenience of observing and comparing. **(F)** Micrographs of TEM. Note the vast amount of dense distributing CMNV-like particles in the necrotic cytoplasm of cells of ommatidium and the nerve tissue of eyestalk. **(F1)** Showed Ecc and Con; **(F2)** showed Crc; **(F3)** showed Ret; **(F4)** showed Mee; **(F5)** showed Sig and Mei. Scale bars were 200 μm in **(D)**; Scale bars were 5000, 1000, 200, and 100 nm in **(F)**. NC, negative control; Mag+, low magnification rate; Mag++, moderate magnification rate; Mag+++, high magnification rate.

The histological examinations showed that the morphology of the epicorneagenous cells, the cone cells, and the crystalline cones tend to become irregular; meanwhile, the boundaries were changed to be blurred between these three types of cells, as well as that of crystalline tracts (Figure 1D1 HE). Micrographs of *in situ* hybridization revealed that the intense purple positive hybridization signals of the CMNV RNA probe presented in the epicorneagenous cells, the cone cells, the crystalline cones, and crystalline tracts (Figure 1D1 ISH). Under the TEM, vacuolation and damage, together with numerous un-enveloped, CMNV-like particles could be observed within the cytoplasm of the epicorneagenous cells and the cone cells (Figure 1F1). Massive amounts of similar spherical CMNV-like particles with a diameter of about 25.3 ± 2.2 nm (*n* = 16) were also present in the cytoplasm of the crystalline cones (Figure 1F2).

**FIGURE 2:**
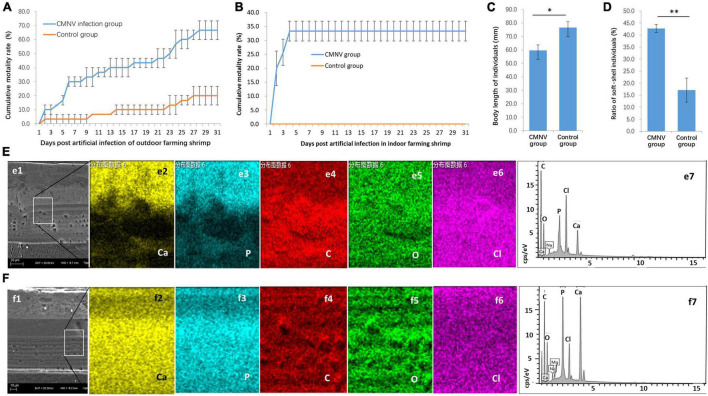
The CMNV artificial challenge test in outdoor and indoor farming shrimp. **(A)** Cumulative mortality curves of *P. vannamei* shrimp in the outdoor artificial challenge test. **(B)** Cumulative mortality curves of *P. vannamei* shrimp in the indoor artificial challenge test. **(C)** Comparing of body length of shrimp individuals from the CMNV infected group and control group at the ending of the challenge test. **(D)** The ratio of soft-shell individuals from the CMNV infected group and control group. **(E)** Determination of calcium element in the carapace of the individual from the CMNV infected group. **(e1)** Micrographs of TEM of the longitudinal section of cephalothorax carapace from shrimp artificially infected with CMNV. **(e2–6)** Micrographs of field emission scanning electron microscopy (FESEM) of the longitudinal section of cephalothorax carapace of the shrimp individuals from the infected group; **(e2–6)** showed the distribution of main constituent elements (including calcium, phosphorus, carbon, oxygen, and chlorine) in the exo-cuticle of cephalothorax carapace. **(e7)** Energy spectrum characteristics in dissociation of main constituent elements in exo-cuticle of cephalothorax carapace of the shrimp individuals from the infected group in the energy-dispersive X-ray spectroscopy (EDS) analysis. **(F)** Determination of calcium element in the carapace of the individual from the control group. **(f1)** TEM of the longitudinal section of cephalothorax carapace of the shrimp individuals in the control group. **(f2–6)** Micrographs of FESEM of the longitudinal section of cephalothorax carapace of the shrimp individuals from the control group; **(f2–6)** showed the distribution of main constituent elements (including calcium, phosphorus, carbon, oxygen, and chlorine) in the exo-cuticle of cephalothorax carapace. **(f7)** Energy spectrum characteristics in dissociation of main constituent elements in exo-cuticle of cephalothorax carapace of the shrimp individuals from the control group in the EDS analysis.

**FIGURE 3:**
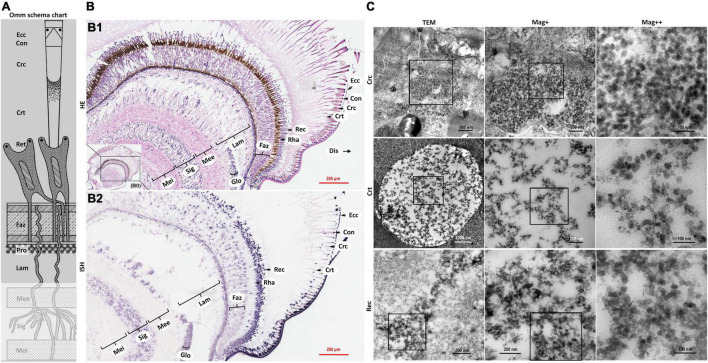
Schematic diagram of ommatidia structure of *P. vannamei*, and micrographs of HE, ISH, and TEM of eyestalk from shrimp artificially infected with CMNV. **(A)** Schematic diagram of ommatidia structure of shrimp *P. vannamei*. Ecc, epicorneagenous cells cytoplasm; Con, cone cells; Crc, crystalline cones; Crt, crystalline tracts; Rec, retinular cell (including the Rha and Ret); Faz, fasciculated zone; Pro, primary optic nerve fibers; Lam, lamina ganglionaris; Mee, medulla externa; Sig, sinus gland; Mei, medulla interna. The same schematic diagram has been included in Figures 1, 4 for the convenience of observing and comparing. **(B1)** Micrographs of H&E staining. Noted the vacuolation, karyopyknosis, and structural failure in almost all types of cells that make up the ommatidium, as well as in the nerve tissue of eyestalk. Ecc, epicorneagenous cells cytoplasm; Con, cone cells; Crc, crystalline cones; Crt, crystalline tracts; Rec, retinular cell; Rha, rhabdom; Faz, fasciculated zone; Lam, lamina ganglionaris; Glo, globuli cell; Mee, medulla externa; Sig, sinus gland; Mei, medulla interna. Note that **(B1)** showed the enlarged view of framed areas in **(B0)**. **(B2)** Micrographs of ISH. Noted the purple hybridization signal at the cells of the ommatidium and the nerve tissue of eyestalk which demonstrated apparent vacuolation. Scale bars were 200 μm in **(B1,B2)**. **(C)** Micrographs of TEM of the several components of the ommatidium for eyestalk of shrimp artificially infected with CMNV. Note the vast number of CMNV-like particles in the necrotic cytoplasm of cells of ommatidium of the eyestalk. Scale bars were 500, 200, and 100 nm in Crc; Scale bars were 1000, 200, and 100 nm in Crt; Scale bars were 500, 200, and 100 nm in Rec. Mag+: low magnification rate; Mag++: moderate magnification rate; Mag+++: high magnification rate.

**FIGURE 4:**
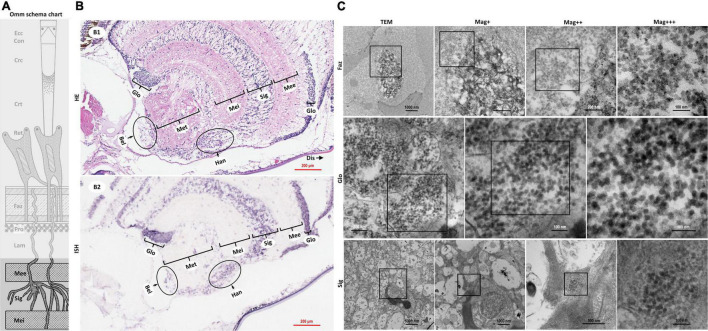
Schematic diagram of ommatidia structure of *P. vannamei*, and micrographs of HE, ISH, and TEM of the nerve tissue and the hormone secretion zone of eyestalk from shrimp artificially infected with CMNV. **(A)** Schematic diagram of ommatidia structure of shrimp *P. vannamei*. Ecc, epicorneagenous cells cytoplasm; Con, cone cells; Crc, crystalline cones; Crt, crystalline tracts; Rec, retinular cell; Faz, fasciculated zone; Pro, primary optic nerve fibers; Lam, lamina ganglionaris; Mee, medulla externa; Sig, sinus gland; Mei, medulla interna. The same schematic diagram has been included in Figures 1, 3 for convenience of observing and the comparing. **(B1)** Micrographs of H&E staining. Noted the vacuolation, karyopyknosis, and structural failure in the nerve tissue and hormone secretion zone of the eyestalk. Glo, globuli cell; Mee, medulla externa; Sig, sinus gland; Mei, medulla interna; Met, medulla terminalis; Han, Hanstrm organ; Bel, organ of Bellonci. Note that **(B1)** showed the enlarged view of partial areas in **(B0)** of Figure 3. **(B2)** Micrographs of ISH. Noted the purple hybridization signal at the cells of the nerve tissue and the hormone secretion zone of eyestalk which demonstrated apparent vacuolation. Scale bars were 200 mm in **(B1,B2)**. Note that **(B2)** was partially overlapping with the **(B2)** of Figure 3. **(C)** TEM of ultrastructural pathology of the ommatidium of and the nerve tissues for eyestalk of shrimp artificially infected with CMNV. Note the vast number of CMNV-like particles in the necrotic cytoplasm of cells of Faz, Glo, and Sig of the eyestalk. Scale bars were 1000, 500, 200, and 100 nm in Faz; Scale bars were 200, 100, and 100 nm in Glo; Scale bars were 5000, 1000, 500, and 100 nm in Sig. Mag+: low magnification rate; Mag++: moderate magnification rate; Mag+++: high magnification rate.

Micrographs of HE staining sections demonstrated that the retinular cell bodies were broken and fractured, and the cytoplasm of retinular cells containing amount pigment granules were tending to be vacuolation (Figure 1D2 HE). A purple hybridization signal was clearly shown in the damaged cytoplasm of the retinular cell in the ISH inspection (Figure 1D2 ISH). TEM examinations proved the presence of CMNV-like particles in the cytoplasm of retinular cells (Figure 1F3).

Severe vacuolation, karyopyknosis, and structural failure in the lamina ganglionaris, as well as mild vacuolation, karyopyknosis, and structural damage in the medulla externa, could be observed in the micrograph of HE staining sections (Figures 1D3 HE,D4 HE). Micrographs of ISH demonstrated that the intense and mild purple positive hybridization signals of the CMNV probe presented in the lamina ganglionaris and the medulla externa, respectively (Figures 1D3 ISH,D4 ISH). Under TEM, myelin sheath stripping around the dendrite of the nerve cell, and vacuolation of cytoplasm in the nerve cell were noticed in the medulla externa. In addition, a vast of CMNV-like particles had been found in the cytoplasm of the nerve cell of the medulla externa (Figure 1F4).

Histopathological changes including karyopyknosis of the hemocytes and nerve cells, more severe vacuolation and structural failure of nerve cells in the sinus gland, as well as mild vacuolation, karyopyknosis and structural damage in the medulla interna, were revealed by the histological examinations of H&E staining sections (Figures 1D5 HE,D6 HE). Meanwhile, the purple positive hybridization signals of the CMNV probe were proved to be present in the corresponding cells and tissue in the sinus gland and the medulla interna by the ISH (Figures 1D5 ISH,D6 ISH). Even more evidence of severe vacuolation, structural failure of nerve cells, and synthesis of CMNV-like particles were revealed in the sinus gland and the medulla interna by the TEM investigation (Figure 1F5).

No positive hybridization signals appeared on the sections from the same samples without the CMNV RNA probe in the hybridization process (Figures 1D1–6).

### The Artificial Challenge of Covert Mortality Nodavirus in Outdoor and Indoor Farming Shrimp

For further confirmation of pathogenicity and pathogenic characteristics of CMNV infection, artificial challenge experiments with low CMNV dose were conducted in outdoor and indoor farming shrimp, respectively. The results of the challenge test in the outdoor farming shrimp showed that the cumulative mortality of shrimp individuals in the CMNV infection group was 66.7% ± 6.7% post 31 days (Figure 2A). In contrast, the cumulative mortality of shrimp individuals was 20.0% ± 6.7% in the control group (Figure 2A). The results of the challenge test in indoor farming shrimp showed that the cumulative mortality of shrimp individuals in the CMNV infection group was 33.3% ± 3.6% post 31 days, and the cumulative mortality was 0.0% in the control group (Figure 2B). A very obvious phenomenon in the challenge test was that the growth rates of the shrimp individuals from the infection and the control groups were significantly different, and shrimp individuals from the infection group grew slower than those from the control group (Figure 2C). The shells of shrimp individuals from the infection group were softer than those from the control group. If lower than two-thirds of the mean value of calcium content in the carapace of the cephalothorax of shrimps in the control group were used as the standard to judge the shell softening individuals of shrimp, the ratio of shell softening individuals from the infected group and the control group were 42.9% and 17.1% (Figure 2D), respectively. The elemental weight percent (%) of calcium in the main constituent elements (including calcium, phosphorus, carbon, oxygen, and chlorine) in the exo-cuticle of cephalothorax carapace of the shrimp individuals from the infected group and the control group was 4.46% and 14.09%, respectively (Figures 2E,F).

In addition, the RT-PCR analysis indicated that the shrimp samples of the infected group were strongly positive for CMNV, whereas shrimp from the control group were negative.

### Microstructural and Ultrastructural Changes Caused by Covert Mortality Nodavirus Artificial Infection

Microstructural and ultrastructural changes in the eyestalk, the ventral nerve cord, and the swimming legs of the shrimp individuals from the CMNV infection group were investigated by using the histopathological, ISH, and TEM methods.

The severe structural failure occurred in almost all types of cells that make up the ommatidium including the epicorneagenous cells, the cone cells, the crystalline cones, the crystalline tracts, the retinular cell, and the rhabdom (Supplementary Figures 2, 5). Vacuolation could be clearly observed in most components of the ommatidium, such as in the crystalline tracts and the rhabdom, in the primary optic nerve fibers of the fasciculated zone, in the nerve and glial fibers of the lamina ganglionaris, as well as in the cytoplasm of the retinular cells. Karyopyknosis were accrued both in the retinular cells and in the cells of the fasciculated zone of ommatidium (Figure 3B HE and Supplementary Figures 2, 5).

Micrographs of ISH demonstrated that the intense purple positive hybridization signals of the CMNV probe presented in the following cells and components covering the epicorneagenous cells, the cone cells, the crystalline cones, the crystalline tracts, the retinular cells, the primary optic nerve fibers in the fasciculated zone, the nerve and glial fibers in the lamina ganglionaris (Figure 3B2 ISH). TEM examinations proved the presence of the vast amount of CMNV-like particles in the cytoplasm of most cell types of the ommatidium, including the crystalline cones, the crystalline tracts, the retinular cells, and the cells in the fasciculated zone (Figures 3C Crc, Crt, and Rec, 4C Faz).

The histological examinations revealed that severe vacuolation, karyopyknosis, and structural failure not only occurred in the nerve enrichment zone like the medulla externa, the sinus gland, the medulla interna, and the medulla terminalis, but also occurred in the hormone secretion zone including the globuli cells, the Hanstrm organ, and the organ of Bellonci (Supplementary Figure 2b). ISH results demonstrated that the intense purple positive hybridization signals of CMNV probe could be observed in the sinus gland, in the Hanstrm organ, and in the organ of Bellonci, as well as in the globuli cells (Figure 4B2). The tissues of the medulla externa, the medulla interna, and the medulla terminalis presented mild hybridization signals of the CMNV probe. The results of TEM analysis indicated that large quantities of CMNV-like particles assembled in the cytoplasm of the globuli cell and the cells of the sinus gland (Figure 4C Glo and Sig). Meanwhile, severe vacuolation of the nerve cells in the sinus gland could be observed in the view of TEM examination.

**FIGURE 5:**
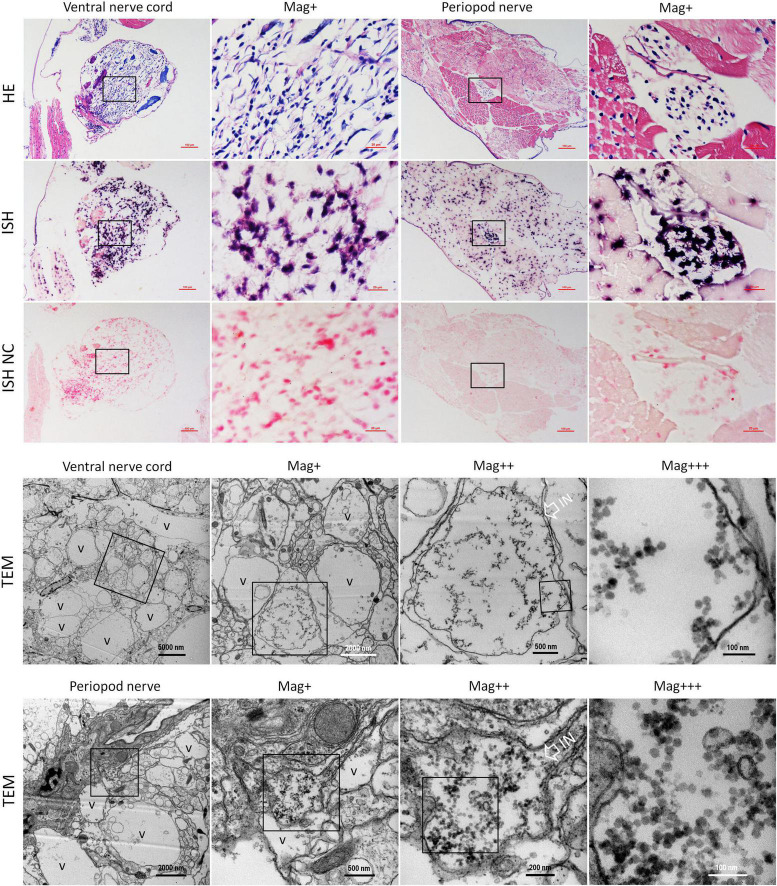
Micrographs of HE, ISH, and TEM of the ventral nerve cord and the pleopods nerve of shrimp artificially infected with CMNV. Note the severe cytoplasmic vacuolation and karyopyknosis in the ventral nerve cord and the pleopods nerve, as well as the extensive muscular lysis, myonecrosis of skeletal muscle in the pleopods. ISH purple hybridization signal of CMNV probe could be observed both in the ventral nerve, as well as in the necrotic skeletal muscle and the nerve cells of pleopods nerve. Note the vacuolar degeneration cytoplasm of nerve cells both in the ventral nerve cord and the pleopods nerve. Note that a vast number of CMNV-like particles are distributed only in the starting or middle-stage vacuolar cytoplasm of nerve cells, no viral particles existed in the cytoplasm of entirely vacuolar nerve cells. IN, inclusion body; NC, negative control; Mag+, low magnification rate; Mag++, moderate magnification rate; Mag+++, high magnification rate; V, vacuolation. Scale bars in the HE and ISH micrographs were 100 and 20 μm; Scale bars were 5000, 1000, 500, and 100 nm in ventral nerve cord TEM micrographs, and were 2000, 500, 200, and 100 nm in the periopod nerve TEM micrographs.

Meanwhile, histological examination of the nerve cord in the abdominal segment showed that the severe cytoplasmic vacuolation and the karyopyknosis occurred in almost all the typical nerve cord cells like the sensory/motor fibers & interneurons, the neurosecretory cells, and the giant cells (Figure 5 HE). ISH results indicated that intense hybridization signals of the CMNV probe were presented in the above necrotic and vacuolar nerve tissues (Figure 5 ISH). The ultrathin sections of the ventral nerve cord revealed severe vacuolation and a large number of CMNV-like particles presented in the cytoplasmic areas of the necrotic neurons. An interesting phenomenon was that mass of CMNV-like particles was distributed in the starting/middle-stage vacuolar degeneration cytoplasm of nerve cells, whereas there were no any CMNV-like particles in the cytoplasm of entirely vacuolar nerve cells (Figure 5 TEM).

In addition, extensive muscular lysis, and myonecrosis of skeletal muscle (Figure 5) could be observed in the swimming legs in the histological examination of pleopods sections of the CMNV infected individuals. Severe cytoplasmic vacuolation and karyopyknosis also occurred in the segmental nerve in the pleopods as it presented in the ventral nerve cord. ISH purple hybridization signal of CMNV probe could be observed both in the necrotic skeletal muscle and in the nerve cells of pleopods segmental nerve. TEM examination results revealed the similar ultrastructural pathological changes of the nerve tissue in swimming legs to that occurred in the nerve cord of the abdominal segment, that is, a vast amount of CMNV-like particles distributed only in the starting or middle-stage vacuolar cytoplasm of nerve cells, no viral particles present in the cytoplasm of entirely vacuolar nerve cells (Figure 5).

No positive hybridization signals appeared on the sections from the shrimp individuals from the control group (Figure 5 ISH NC and Supplementary Figure 6).

### Differential Transcriptome Analysis of the Healthy and Infected Shrimp Individuals

Furthermore, with the help of Gene Ontology (GO), Kyoto Encyclopedia of Genes and Genomes (KEGG) pathway analyses, we investigated the molecular mechanism of the difference in growth rate between the healthy and the CMNV infected shrimp individuals. Firstly, based on the 300 million (300,557,286) raw reads, a total of 299.6 million (299,659,796) clean reads were produced by filtering the low-quality data (Supplementary Table 2). Both the statistics of the bases from the clean data and Ribosomal comparing statistics of the sequenced reads indicated that the clean data were of high quality (Supplementary Tables 3, 4).

**FIGURE 6:**
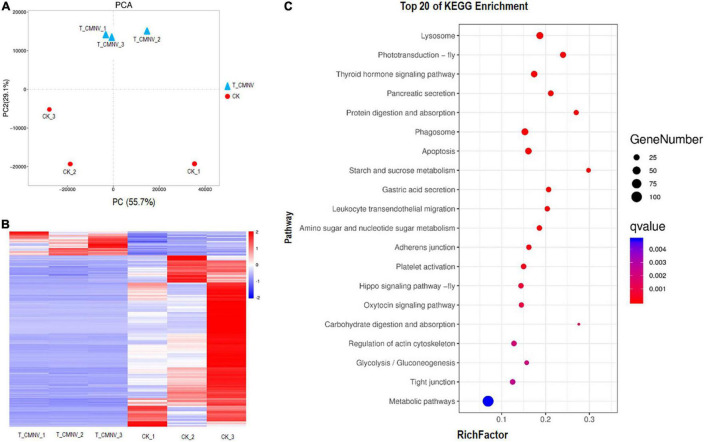
Differential transcriptome analysis of the healthy and infected shrimp individuals. **(A)** Principal component analysis (PCA) of the global gene expression of the healthy and infected shrimp individuals. Triangles indicate the infected shrimp individuals (*n* = 3, T_CMNV) and dots indicate the healthy shrimp individuals (*n* = 3, control check, CK). Overall gene expression is driven by the CMNV infection. **(B)** Heat map showing log10 gene expression ratios of the healthy and CMNV infected shrimp. Genes with similar expression patterns were clustered. The intensity of the color indicates gene expression levels that were normalized according to log10 (FPKM + 1) values. The calculated *p*-value was gone through FDR Correction, taking FDR ≤ 0.05 as a threshold. The up-regulated genes are shown in red and the down-regulated genes are shown in blue. **(C)** KO enrichment analysis for the DEGs between the healthy and CMNV infected shrimp. The vertical axis label represents the pathway, and the horizontal axis label represents the rich factor. Circles indicate the numbers of enriched genes and colors depict the *P*-value. The result of the KEGG pathway analysis of DEG showed that DEGs were enriched into different GO terms. Significant enrichment was observed in the regulation of lysosome, photo-transduction-fly, thyroid hormone signaling pathway, pancreatic secretion, phagosome, et al., which were strongly associated with digestion, absorption, growth hormone, and visual light conduction.

Principal component analysis (PCA) was performed to compare the global gene expression of the healthy and infected shrimp individuals. The result of PCA indicated that samples of CMNV infection clustered tightly and were separated from the control check (CK) (Figure 6A), indicating that infection with CMNV altered the overall GE profile thoroughly.

The heat map was constructed to visually show all the DEG of the healthy and CMNV infected shrimp (Figure 6B). In order to obtain a global view of the change in shrimp gene expression between the healthy and CMNV infected shrimp, one paired comparison (CK vs. T_CMNV) was performed. In all, 198 up-regulated genes and 1,415 down-regulated genes were detected in the RNA-seq analysis at significantly different levels (FDR < 0.05 and | log2FC| > 1) (Supplementary Figures 7, 8).

After GO enrichment analysis, DEGs were enriched into different GO terms. The top significant enrichment was observed in terms including collagen metabolic process, multicellular organism metabolic process, sensory perception of light stimulus, sensory perception, and neurological system process (Supplementary Figure 9). The KEGG database was used to analyze pathways in order to further define DEG function in the shrimp. The result of the KEGG pathway analysis of DEG showed that DEGs were enriched into different KEGG pathways and the top 20 enrichment KEGG pathways with FDR < 0.05 in the healthy and CMNV infected shrimp were listed (Figure 6C). The top significant enrichment was observed in the regulation of lysosome, photo-transduction-fly, thyroid hormone signaling pathway, pancreatic secretion, phagosome, et al., which were strongly associated with the metabolic process including digestion, absorption, growth hormone, as well as the sensory perception and neurological system process like visual light conduction (Figure 6C).

### Validation of Differentially Expressed Genes With qRT-PCR

To verify the DEGs detected by RNA-seq, the expressions of 4 DEGs related to the phototransduction, phagosome, thyroid hormone signaling pathway, and pancreatic secretion metabolic pathways were selected and tested by using qRT-PCR from the top 20 KEGG enrichment genes. The results showed that the expression patterns of these DEGs in the healthy and infected shrimp individuals were consistent with that of the RNA-seq trend. Compared with the control group, three DEGs, i.e., LOC113803353 (phototransduction-related gene), LOC113806545 (phagosome related gene), and LOC113810077 (pancreatic secretion metabolic pathways related gene) were downregulated, and one DEG, i.e., LOC113808838 (thyroid hormone signaling pathway) was upregulated (Supplementary Figure 10).

## Discussion

Covert mortality nodavirus was a new member in the genus *Alphanodavirus*, the target tissues/organs and histopathology of CMNV infection in shrimp had not been studied thoroughly yet. A previous report just studied the susceptibility of CMNV in the hepatopancreas, muscle, and lymph organs in the shrimp, whereas the CMNV susceptibility in more organs of shrimp had not been known yet. The present study revealed that nerve tissues distributing both in the eyestalks, abdominal segment and pleopods were also the important target tissue of CMNV infection. Previous preliminary studies demonstrated that CMNV infection could result in cytoplasm vacuolation of the hepatopancreocytes, multifocal myonecrosis of the striated muscle, hemocytic infiltration, and karyopyknosis of hemocytes in the target tissues, as well as the presence of the eosinophilic inclusions in the tubular epithelium of hepatopancreas and lymphoid spheroids (Zhang et al., 2014, 2017). The present study observed that both in the natural and artificial infected white leg shrimp individuals, the similar histological damages as previous reports occurred also in the hepatopancreas and muscles (Data not shown). More importantly, severe vacuolation and damage, as well as the vast amount of CMNV-like particles were observed for the first time in the nerve tissues both from the eyestalk, the pleopods, and the ventral nerve cord of the CMNV infected individuals in this study.

**FIGURE 7:**
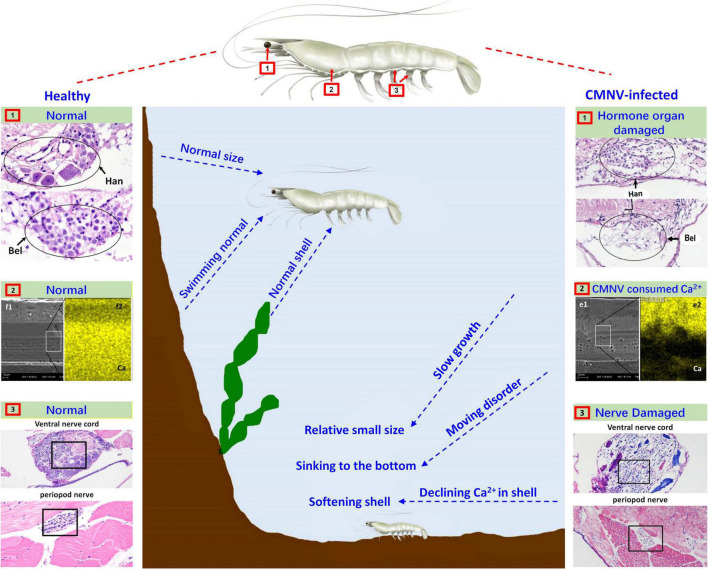
Schematic diagram of the pathogenic mechanism of CMNV infection in *P. vannamei*. Figures in the left showed the hanstrm organ (Han) and Bellonci (Bel) organ **(1)**, ventral nerve cord and periopod nerve **(2)**, TEM, and FESEM of the longitudinal section of cephalothorax carapace **(3)**, of the shrimp individuals in the control group. Figures in the middle part described the clinical symptoms and behavioral characteristics of healthy shrimp and CMNV-infected shrimp. Figures in the right showed the hanstrm organ (Han) and Bellonci (Bel) organ **(1)**, ventral nerve cord and periopod nerve **(2)**, TEM and FESEM of the longitudinal section of cephalothorax carapace **(3)**, of the shrimp individuals in the CMNV infection group. The severe cell damage of the Han and Bel **(1)**. The severe pathological damages in the nerve cells of both the ventral nerve cord and segmental nerve of the pleopods **(2)**. The obvious lower calcium content (the more yellow means more calcium) in the FESEM figure of **(f2)** contrasting with the health one in the left figure of **(e2) (3)**.

In the eyestalk, the primary optic nerve fibers, nerve, and glial fibers were mainly distributed in the tissues of the fasciculated zone, the rhabdom, the lamina ganglionaris, the medulla externa, the medulla interna, and the sinus gland. The nerve and glial fibers were closely related to the visual signal transduction in the eyestalk of crustaceans (da Silva et al., 2001; Gunawardene et al., 2002; Nesbit et al., 2015), so the vacuolating necrosis of these nervous related tissues would unquestionably result in the decrease of the vision of diseased shrimp individuals. Meanwhile, serious necrosis in the pleopods nerve and the ventral nerve cord, as well as severe skeletal muscle destruction in the pleopods, might correspondingly decrease the swimming performance of diseased shrimp individuals. With comprehensive consideration of the descend both the vision and the swimming performance in diseased shrimp individuals, it would be easy to understand the reason why most of the CMNV infected individuals were hidden on the bottom of farming ponds and then died gradually under that covert environment (Figure 7).

In crustaceans, molting was controlled by the neuropeptides of molt-inhibiting hormone (MIH) and ecdysone (Diwan, 2005; Zhang et al., 2019). A major source of neuropeptides was the X-organ–sinus gland (XO–SIG), and X-organ was an integral part of the hanstrm organ (Han) in crustaceans (Arvy et al., 1954; Loredo-Ranjel et al., 2017; Head et al., 2019). The histological and ultrastructural examination in this study indicated that severe pathological damages occurred in almost all the cells that constitute the ommatidium of the eyestalk, especially in the cells composing ecdysone synthesis-related tissues (hanstrm organ and sinus gland). Even more, the ultra-microstructural observation proved the presence of mass CMNV-like particles in the cytoplasm of several components of the eyestalk, including epicorneagenous cells, cone cells, crystalline cones, retinular cells (Rec), fasciculated zone, globuli cells, and sinus gland. Hence, it could be deduced that the severe histological damages in the related tissues of ecdysone synthesis might result in decreasing the secretion of neuropeptides, and finally reduce the growth rate of the diseased shrimp individuals (Figure 7).

Divalent metal ions were components of numerous icosahedral virus capsids (Trask and Dormitzer, 2006; Xu et al., 2017; Ren et al., 2018; Tarasova and Nerukh, 2018). Calcium ions were found to be vital to the viral capsid structure of the virus member in the family Nodaviridae, as well as the biology of virus-host interactions (Wu et al., 2008; Ho et al., 2018). 240 calcium ions were needed to be incorporated per viral capsid in assembling of noda viruses such as Flock House virus (FHV) and *Macrobrachium rosenbergii* nodavirus (MrNV) (Banerjee et al., 2010; Jariyapong et al., 2014; Ho et al., 2018). The feature of the vast amount of calcium ions consumed in viral capsid assembly of the family Nodaviridae clued that the mass CMNV assembly in the diseased shrimp would consume a huge amount of calcium ions in the stage of acute infection of the virus as well. The result was that the carapace synthesis of diseased shrimp might not be able to obtain enough calcium ions, which led to softening of the diseased shrimp carapace, as well as molting difficulty and ultimately affecting the growth rate of the CMNV infected shrimp individuals (Figure 7).

The microsporidian Enterocytozoon hepatopenaei (EHP) was first described in Thailand in 2009 and it caused hepatopancreatic microsporidiosis (HPM) in farmed black tiger shrimp Penaeus monodon (Tourtip et al., 2009; Tangprasittipap et al., 2013). It was suspected that EHP infection was related to growth retardation in farming shrimp in previous reports (Biju et al., 2016; Salachan et al., 2017). Recently the information from shrimp farmers indicated that it was associated with significant growth retardation that was not clearly noticeable until 2–3 months of cultivation, whereas HPM was not normally associated with shrimp mortality (Salachan et al., 2017; Karthikeyan and Sudhakaran, 2019). During the past 5-years epidemiological investigation of farming shrimp, we found that the diseased shrimp suffering merely EHP infection did not show syndromes of carapace softening. So, both syndromes of slowing growth and carapace softening were the typical characteristic of CMNV acute infection, which was different from the EHP infection.

Up to now, three nodavirus, including MrNV, *Penaeus vannamei* nodavirus (PvNV), and CMNV, had been isolated in crustaceans (Qian et al., 2003; Bonami et al., 2005; Poulos et al., 2006; Tang et al., 2007; Flegel, 2012). These three viruses showed different virulence to hosts. MrNV infection could cause 100% mortality of post-larval and juvenile of *M. rosenbergii* (Arcier et al., 1999; Sahul Hameed et al., 2004; Citarasu et al., 2019). PvNV could cause the survival of pond farming *P. vannamei* to decrease from an average of 74 to 50%, but was not lethal to the *P. vannamei* in laboratory artificial infections (Tang et al., 2007, 2011). The cumulative mortality of *P. vannamei* was up to 84.85% in artificial CMNV infection experiments via *per os* infection (Zhang et al., 2014). In the earth ponds attacked by VCMD, chronic constant death of shrimp could be observed during daily management throughout the farming period, and cumulative mortality of *P. vannamei* might be up to 80% finally (Zhang et al., 2014, 2017). In our previous epidemiological survey of VCMD, we found that there was a relative difference in the mortality of indoor cultured shrimp and outdoor pond cultured shrimp infected with CMNV. Therefore, in order to simulate the shrimp mortality after CMNV infection in different farmed modes (indoor and outdoor), we arranged the outdoor and indoor challenged trials in this study. A low dose of CMNV about 2.0 × 10^4⋅5^ copies per mg shrimp weight was used for viral injecting infection in the present study. During the 31-days artificial infection experiment of outdoor and indoor farming shrimp, the cumulative mortality of individuals in the infection group was 66.7 and 33.3%, respectively, which were similar to the relative difference in the mortality of indoor cultured shrimp and outdoor pond cultured shrimp infected with CMNV. Whereas, the cumulative mortality of individuals in the infection group in outdoor challenged trials was lower than that in our previous report. We deduced that the lower cumulative mortality might be related to the low starting infecting CMNV dose in the challenged group. Meanwhile, the cumulative mortality of shrimp in the infection group in indoor farming was significantly lower than that of shrimp in the infection group in outdoor farming, which indicated that the lethal capacity of CMNV was related to the farming environment, and the stable farming environment might be conducive to reducing the mortality of shrimp caused by CMNV infection.

Because of belonging to the same genus *Alphanodavirus* in the classification, MrNV, PvNV, and CMNV showed similar tissue tropism to a certain degree, that is, all the three viruses could infect the myocyte and cause myonecrosis of skeletal muscle (NaveenKumar et al., 2013; Senapin et al., 2013). Whereas, till now no evidence showed that MrNV and PvNV could infect the nerve tissues. The present study found that CMNV could seriously infect the nerve tissue in the eyestalk, pleopods, and the ventral nerve cord in both the natural and artificial conditions, that is, CMNV distinctly showed neurotropic characteristics, which was very rare for the alphanodaviruses that infect crustaceans. It is well known that the betanodaviruses were neurotropic and could cause “viral nervous necrosis” or “viral encephalopathy and retinopathy” associated with behavioral abnormalities and high mortalities in larvae, juvenile or adult marine fish (Ucko et al., 2004; Walker and Winton, 2010; King et al., 2011; Kim et al., 2019). Hence, the neurotropic characteristics of CMNV were similar with that of betanodaviruses.

It is being increasingly recognized that transcriptome analysis is an efficient tool for characterizing the molecular basis of host–virus relationships (Luciani et al., 2012; Radford et al., 2012; Palesch et al., 2018; Mbulaiteye and Prokunina-Olsson, 2019). Recently, several studies have reported on differential transcriptome analysis in Cherax quadricarinatus hepatopancreas infected with Decapod iridescent virus 1 (DIV1) (Yang et al., 2019), in *Macrobrachium nipponense* (Zhao et al., 2018) and *P. vannamei* infected with white spot syndrome virus (WSSV) (Peruzza et al., 2019). Transcriptome analysis of the healthy and infected shrimp individuals demonstrated that the significant difference in the expression of genes could be induced by the CMNV infection. In KEGG enrichment analysis, significant enrichment of DEG was observed in regulatory pathways of the lysosome, photo-transduction-fly, thyroid hormone signaling pathway, pancreatic secretion, phagosome, et al., and these metabolic pathways were strongly associated with biological processes including the digestion, absorption, and growth hormone. In the present study, the significant down-regulated expression of genes related to digestion, and absorption in the infected shrimp was closely related to the hepatopancreas lesions which were the typical clinical symptoms of CMNV infected shrimp. The significant decline of gene expression of the thyroid hormone signaling pathway was obviously caused by the destruction of the hormone secretion zone including the globuli cells, the Hanstrm organ, and the organ of Bellonci. That is, the destruction and disabling of the tissues including the hepatopancreas, as well as the nerve system in eyestalk and abdominal nerve cord, resulting in the significant down-regulated expression of genes of digestion, absorption, and growth hormones in the CMNV infected shrimp, and finally lead to the slow growth of the CMNV infected shrimp individuals. Hence, the differential transcriptome analysis along with the histopathological and ultra-histopathological analysis in this study revealed the possible molecular mechanism of the slow growth of *P. vannamei* caused by CMNV infection.

In summary, the present study discovered the unique characteristic of neurotropism of CMNV, a new virus of alphanodavirus for the first time. The investigation revealed the pathogenesis of slow growth and mortality of *P. vannamei* induced by CMNV infection under poor environmental conditions, as well as the possible molecular mechanism of the slow growth caused by CMNV infection. The results of this study will be helpful for related practitioners and researchers in finding out strategies to prevent and control VCMD effectively in cultured shrimp.

## Data Availability Statement

Data available on request from the authors.

## Author Contributions

QZ designed the experiments and contributed to naturally infected shrimp sampling. QZ and SL executed the experiments, analyzed the samples, and wrote the manuscript. QZ, JX, YT, SL, XuL, and LY conducted the artificial challenge experiment and sampling. JK supplied the SPF shrimp. SL prepared the histological sections and conducted the *in situ* RNA hybridization assay. XiL, TX, and WW did the molecular and biological analysis and the sequencing work. QZ and LY conducted the TEM and FESEM assay. QZ and WW contributed to the diagram drawing of the shrimp. All authors interpreted the data, critically revised the manuscript for important intellectual contents, and approved the final version.

## Conflict of Interest

The authors declare that the research was conducted in the absence of any commercial or financial relationships that could be construed as a potential conflict of interest.

## Publisher’s Note

All claims expressed in this article are solely those of the authors and do not necessarily represent those of their affiliated organizations, or those of the publisher, the editors and the reviewers. Any product that may be evaluated in this article, or claim that may be made by its manufacturer, is not guaranteed or endorsed by the publisher.

## Footnotes

1
https://sg.idtdna.com/pages/tools/primerquest
