# Supplementary material for: Investigation of Pathogenic Mechanism of Covert Mortality Nodavirus Infection in Penaeus vannamei
Source: Front Microbiol. 2022 May 31;13:904358. doi: 10.3389/fmicb.2022.904358 (PMC9195102; doi:10.3389/fmicb.2022.904358)
Supplement: Supplementary file 1 [file Data_Sheet_1.docx]

Supplementary Material

# Supplementary Figures and Tables

## Supplementary Figures

**
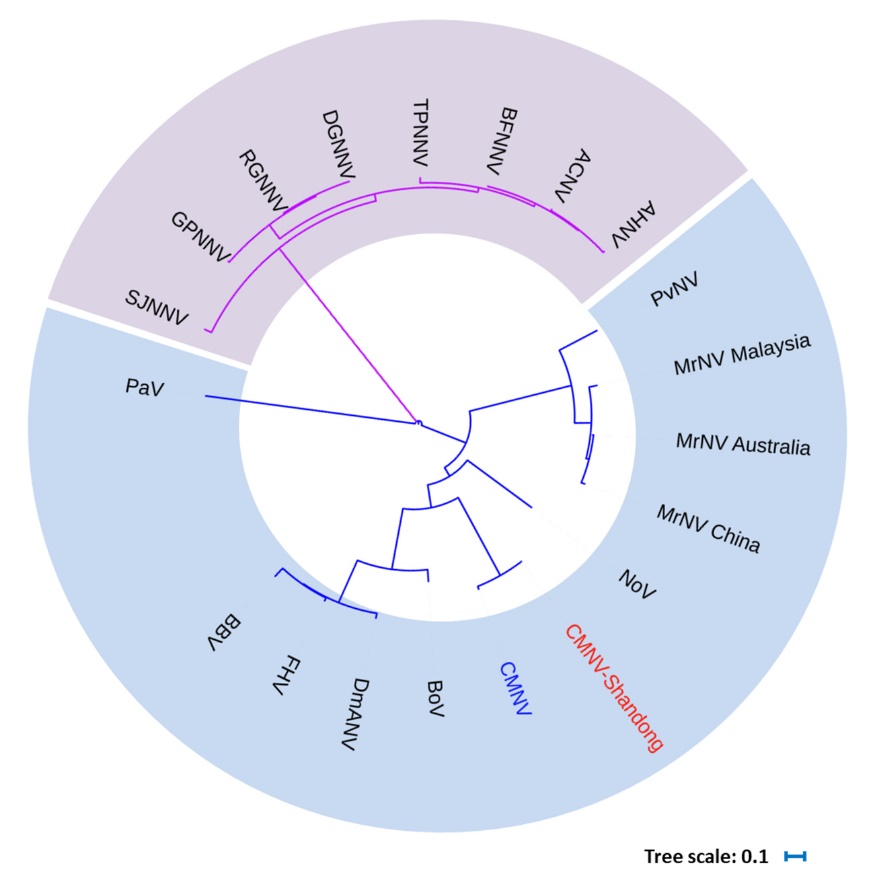
**

**Supplementary Figure 1. Phylogenetic analysis performed using deduced amino acid sequences of RNA-dependent RNA polymerase (RdRp) genes from the diseased white leg shrimp *Penaeus vannamei* (CMNV_Shandong) and other nodaviruses (For virus abbreviations see S1 Table).** The CMNV_Shandong was labeled in red color. A*lphanodavirus* genus was indicated by baby blue background. *Betanodaviruses* genus was indicated by lavender background. The tree was constructed using MEGA software with the maximum-likelihood method. Bootstrap values were calculated with 1,000 replicates of the alignment. Percentage bootstrap values (1,000 replicates) >80% are shown.

**
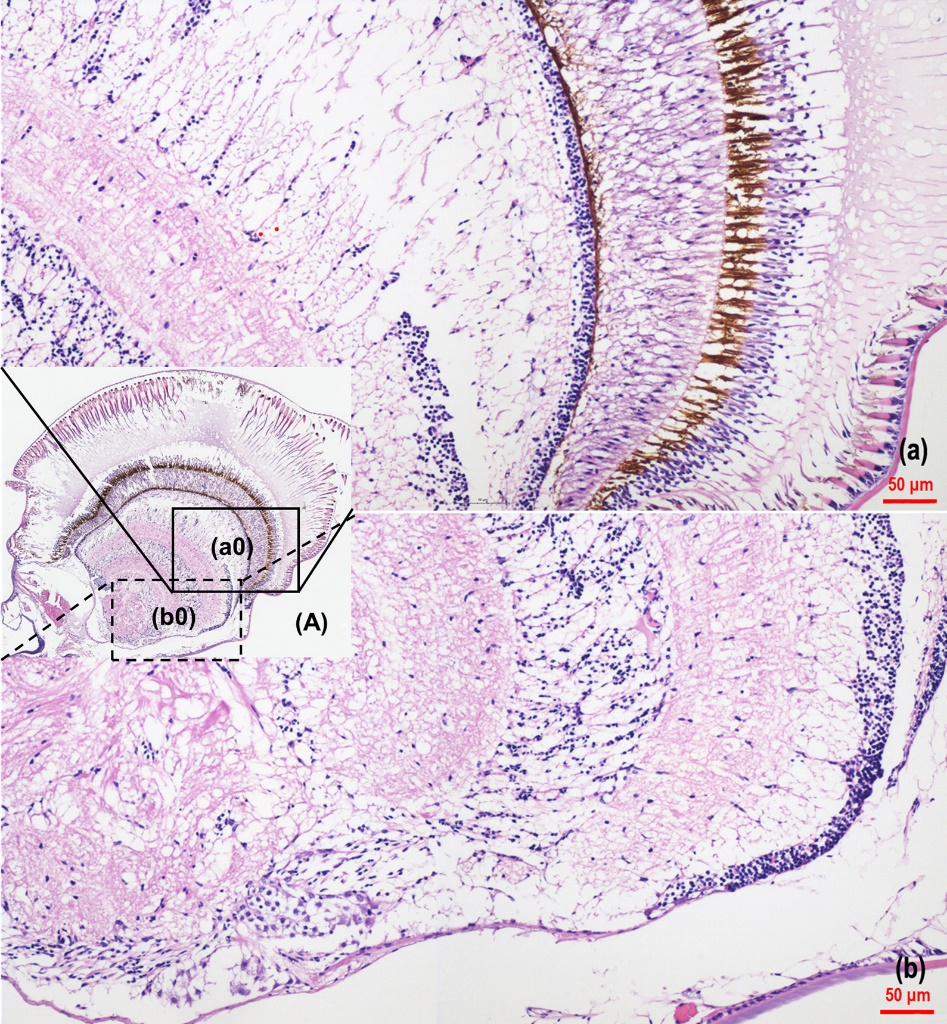
**

**Supplementary Figure 2.** **Micrographs of H&E staining for eyestalk of shrimp artificially infected with CMNV.** (a) Micrographs of H&E staining of the ommatidiums zones. It can be noted that severe structural damage occurred in almost all types of cells that making up the ommatidium. Note the vacuolation in the crystalline tracts (Crt), and rhabdom (Rha), in the primary optic nerve fibers offasciculated zone (Faz), in the nerve and glial fibers (Ngf) of lamina ganglionaris (Lam), as well as in the cytoplasm of retinular cells (Ret). Note the karyopyknosis in the retinular cells (Ret) and the cells offasciculated zone (Faz). (b) Micrographs of H&E staining of the nerve enrichment zone of eyestalk. Note the severe vacuolation, karyopyknosis, and structural failure in the medulla externa (Mee), the sinus gland (Sig), the medulla interna (Mei), and the medulla terminalis (Met), as well as in the hormone secretion zones including the globuli cells (Glo), the Hanstrm organ (Han), and the organ of Bellonci (Bel). Scale bars were 50 μm in (a) and (b). S2 Fig (a) and S2 Fig (b) were the partial enlarged view of ommatidium zones shown in the in S2 Fig. (A), Fig. 3 (B0) & (B1), Fig. 4 (B0) & (B1).

**
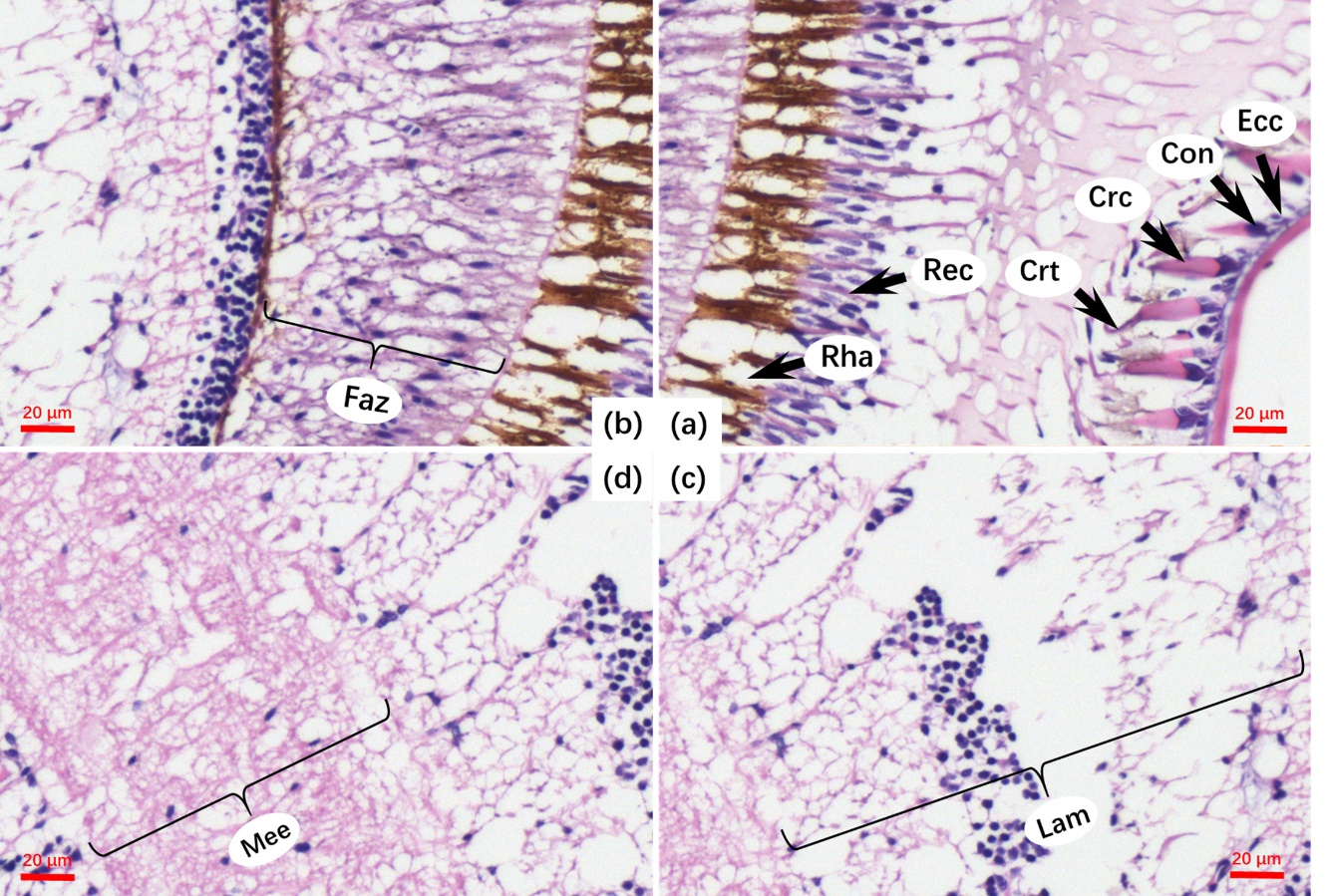
**

**Supplementary Figure 3. Micrographs of H&E staining for** [**partial**](javascript:;) **eyestalk of shrimp artificially infected with CMNV.** (a-b) Micrographs of H&E staining of the ommatidiums zones. It can be noted that severe structural damage occurred in almost all types of cells that making up the ommatidium. Note the vacuolation in the crystalline tracts (Crt), and rhabdom (Rha), in the primary optic nerve fibers offasciculated zone (Faz), as well as in the cytoplasm of retinular cells (Rec). Note the karyopyknosis in the retinular cells (Rec) and the cells of fasciculated zone (Faz). (c-d) Micrographs of H&E staining of the [partial](javascript:;) nerve enrichment zones. Note the severe vacuolation of the nerve and glial fibers in both of the lamina ganglionaris (Lam) and the medulla externa (Mee). Scale bars were 20 μm in (a-d). All the figures in S3 Fig. were the partial enlarged view of ommatidium zones shown in the Fig. 3 (B0) & (B1), Fig. 4 (B0) & (B1).

**
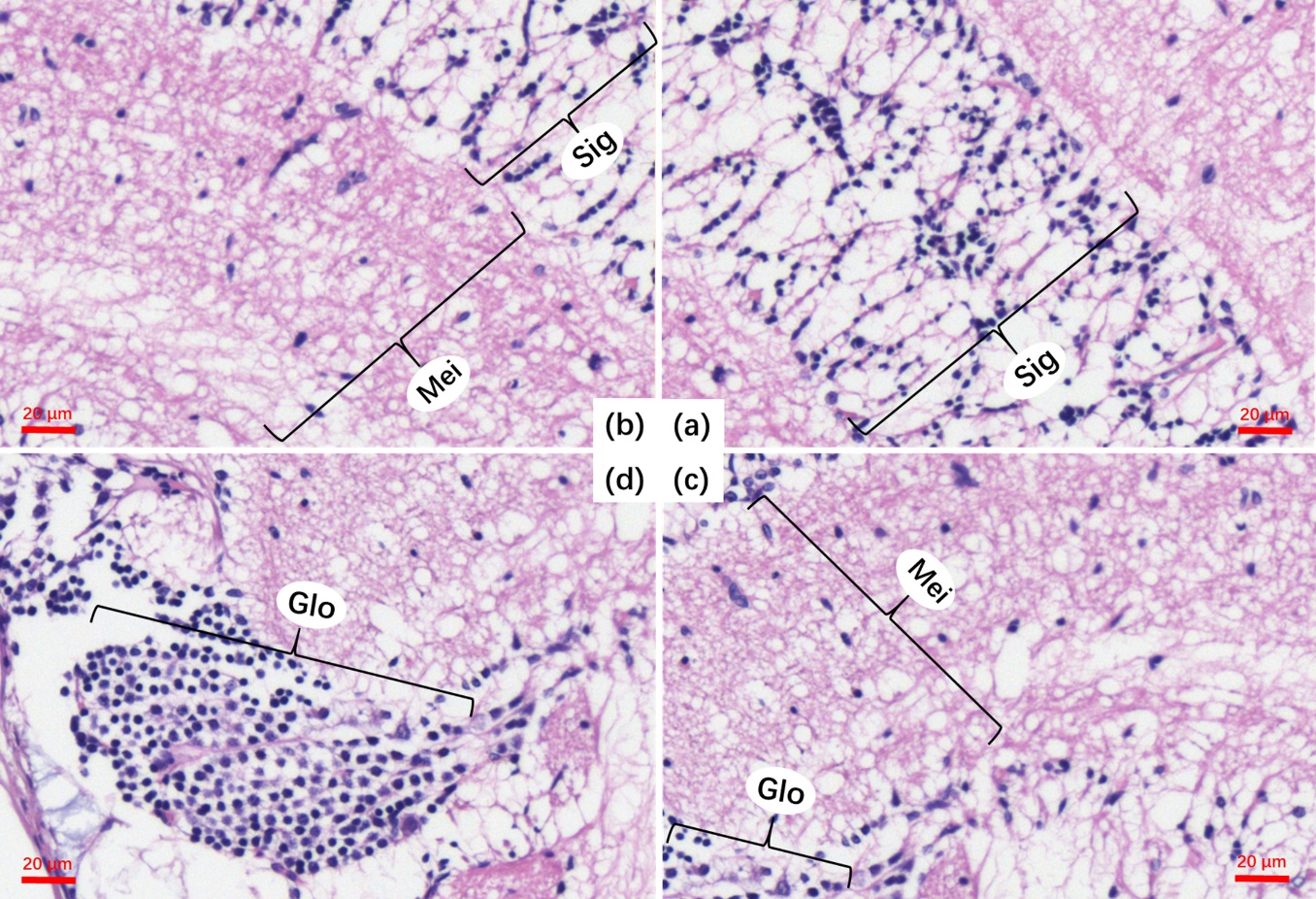
**

**Supplementary Figure 4. Micrographs of H&E staining for** [**partial**](javascript:;) **eyestalk of shrimp artificially infected with CMNV.** (a-d) Micrographs of H&E staining of the [partial](javascript:;) nerve enrichment zones. It can be noted that severe vacuolation, karyopyknosis, and structural failure in the sinus gland (Sig), and the medulla interna (Mei), as well as in the globuli cells (Glo). Scale bars were 20 μm in (a-d). All the figures in S4 Fig. were the partial enlarged view of ommatidium zones shown in the Fig. 3 (B0) & (B1), Fig. 4 (B0) & (B1).

**
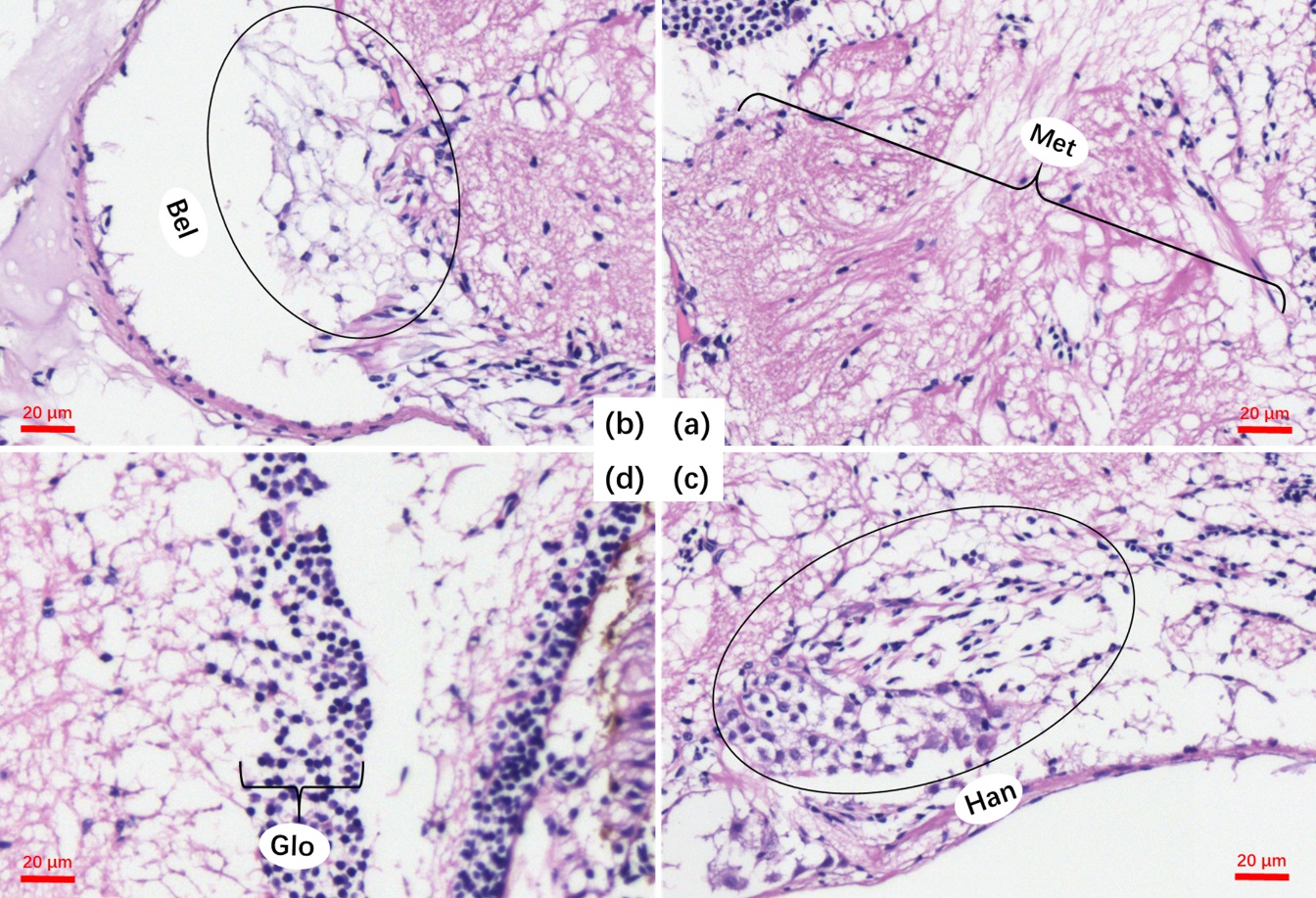
**

**Supplementary Figure 5. Micrographs of H&E staining for** [**partial**](javascript:;) **eyestalk of shrimp artificially infected with CMNV.** (a-d) Micrographs of H&E staining of the [partial](javascript:;) nerve enrichment zones. Note the severe vacuolation, karyopyknosis, and structural failure in the medulla terminalis (Met), as well as in the hormone secretion zones including the globuli cells (Glo), the Hanstrm organ (Han), and the organ of Bellonci (Bel). Scale bars were 20 μm in (a-d). All the figures in S5 Fig. were the partial enlarged view of ommatidium zones shown in the Fig. 3 (B0) & (B1), Fig. 4 (B0) & (B1).

**
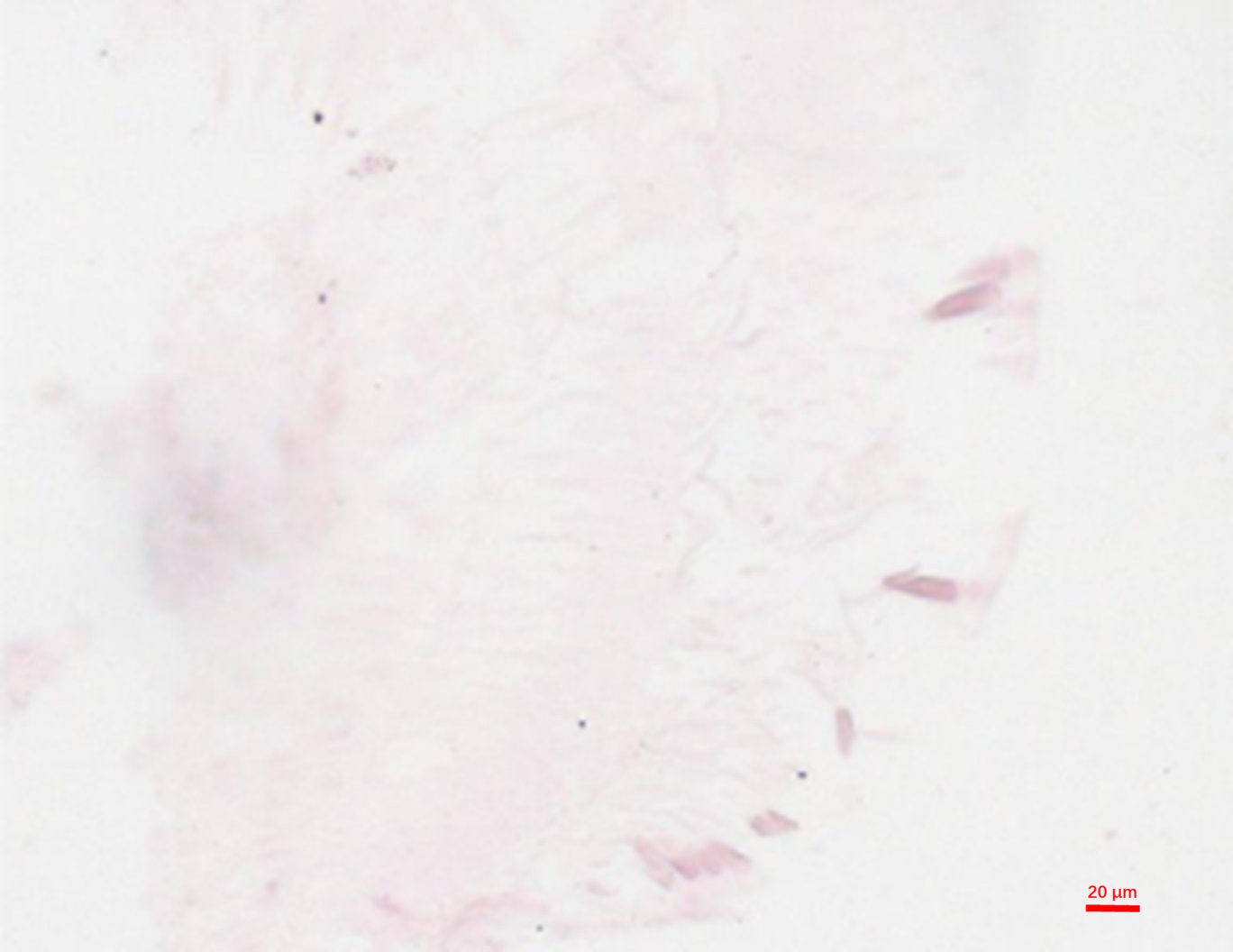
**

**Supplementary Figure 6.** **Micrographs of negative controls of *in situ* hybridization (ISH) without RNA probe for the eyestalk of shrimp artificially infected with CMNV.** Scale bars were 20 μm.

**
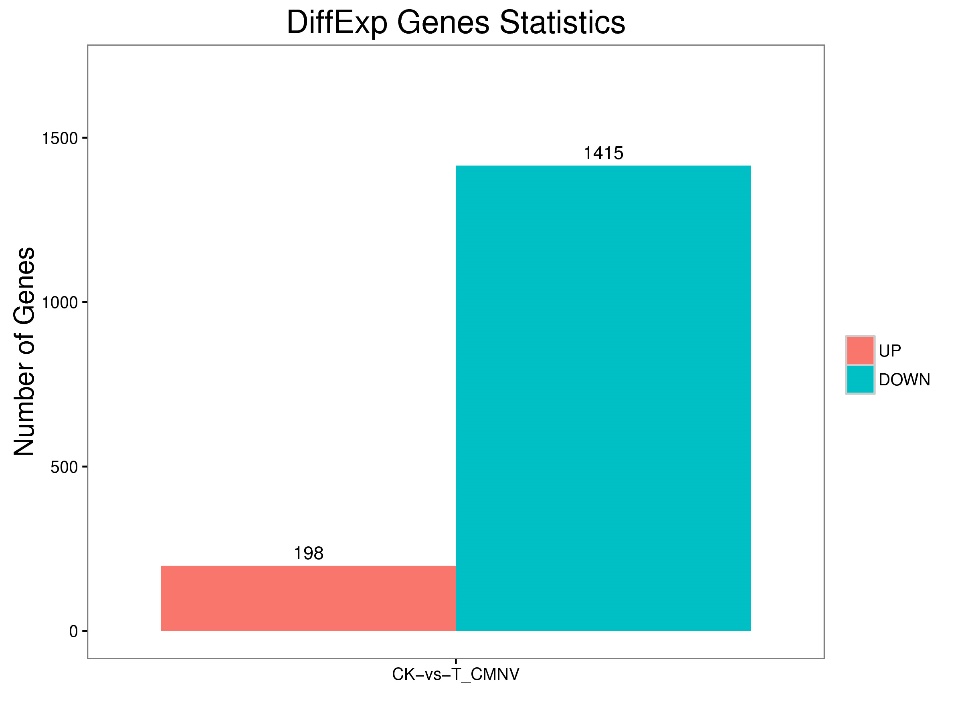
**

**Supplementary Figure 7.** **Differential expressed genes caused by CMNV infection.** The horizontal axis represents the up-regulated/down-regulated genes, and the vertical axis represents the number of genes. The red dots mean significantly up-regulated genes and the green dots represent significantly down-regulated genes.

**
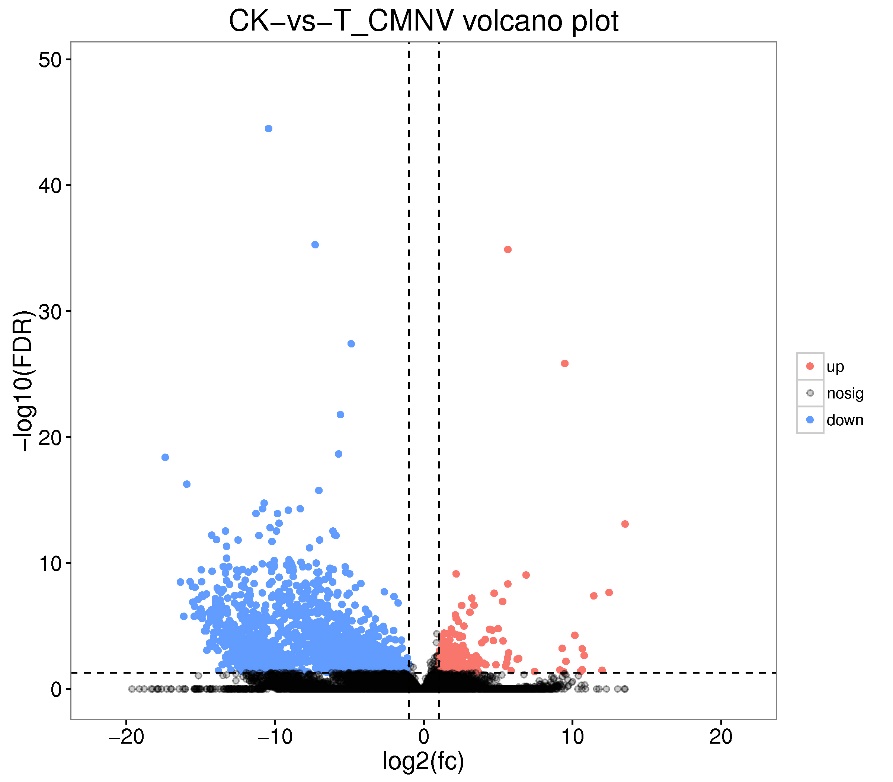
**

**Supplementary Figure 8. Differential expressed genes caused by CMNV infection.** The horizontal axis represents fold changes of gene expression, and the vertical axis represents the statistically significance level. The red dots mean significantly up-regulated genes and the blue dots represent significantly down-regulated genes.

**
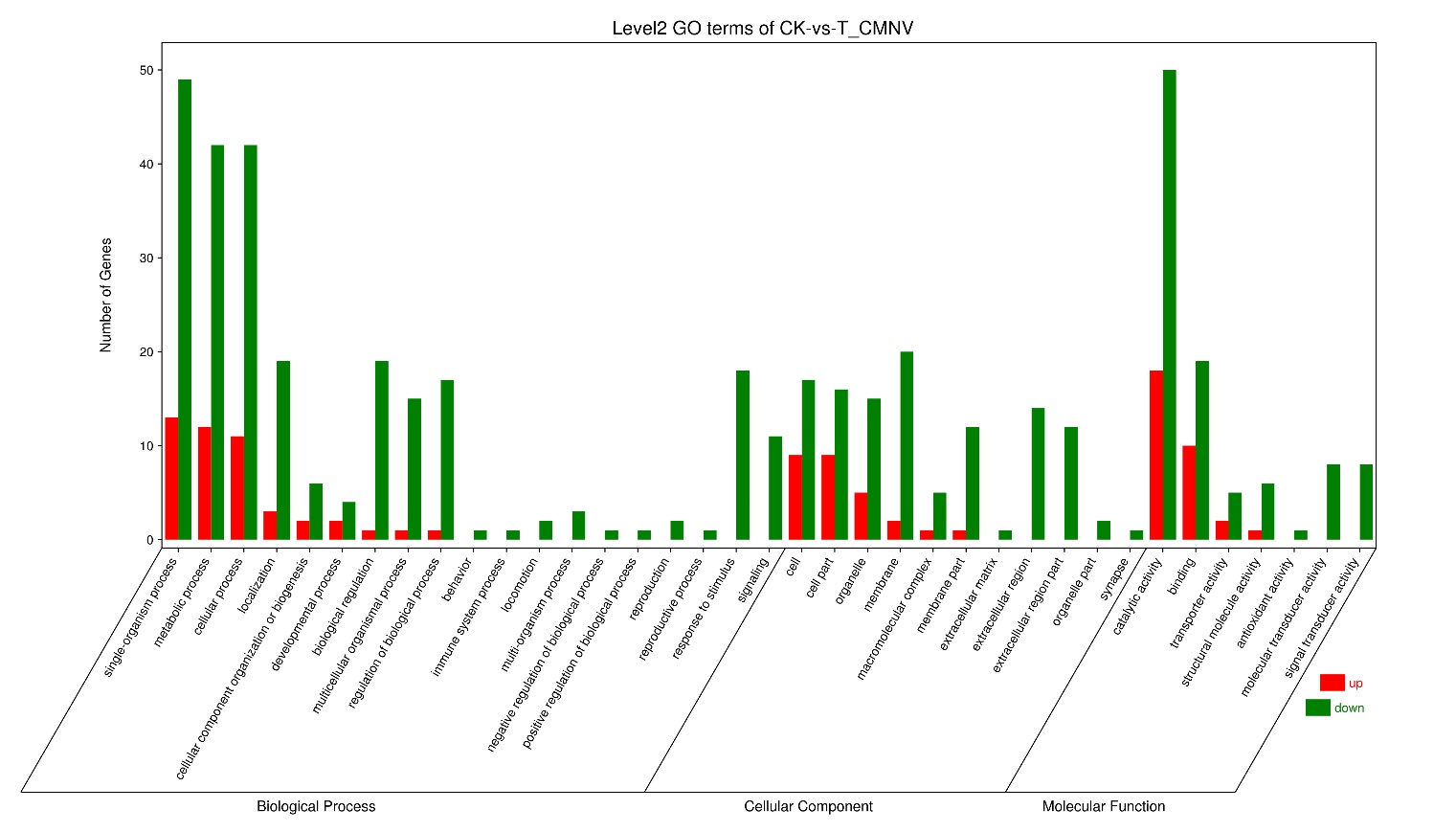
**

**Supplementary Figure 9. Go enrichment analysis for the DEGs between the healthy and CMNV infected shrimp.** The abscissa is the Level 2 GO term, and the ordinate is the number of genes in the term. In all graphs, green dots and bars represent significantly upregulated genes and red dots and bars represent significantly downregulated genes.


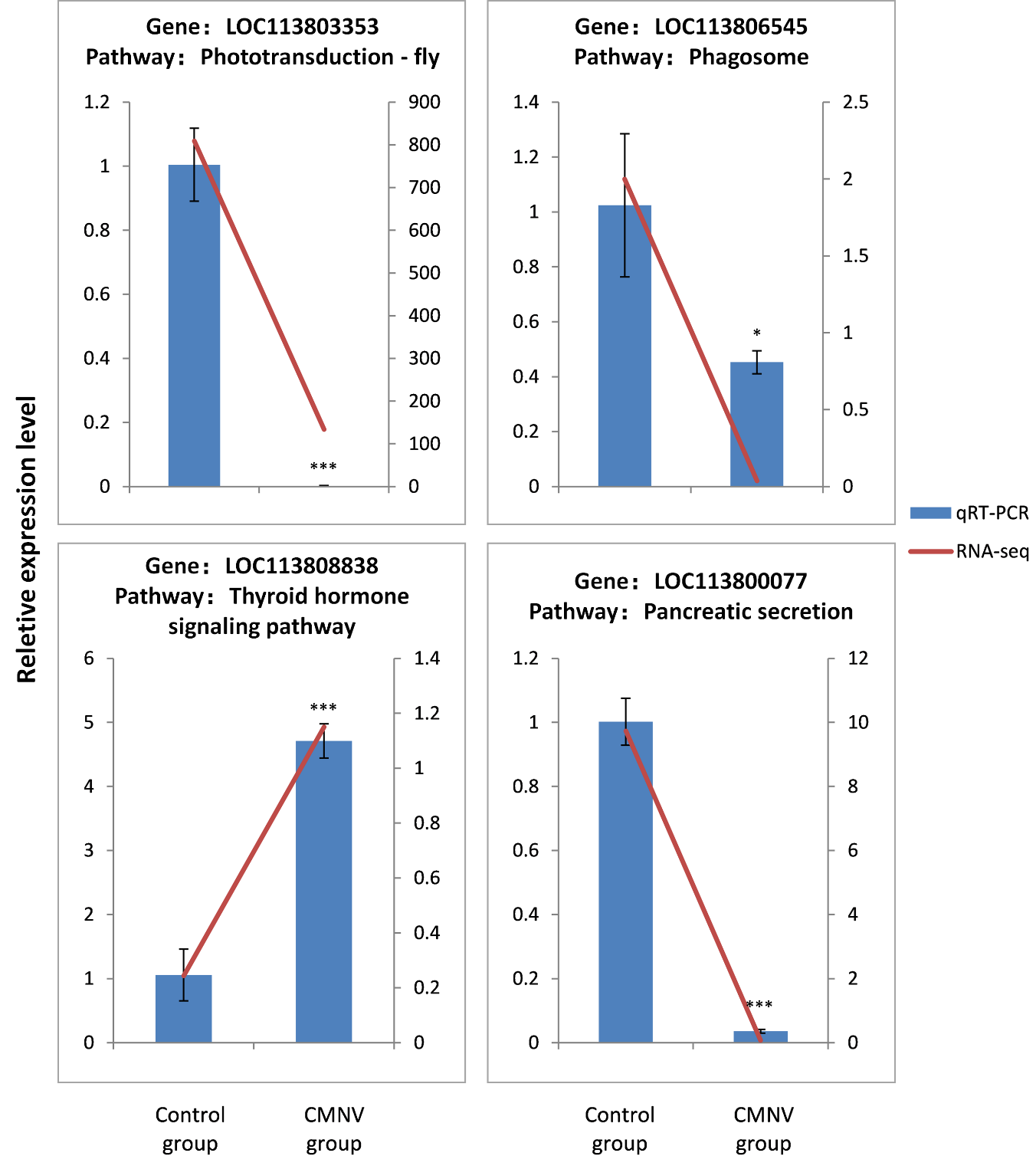


**Supplementary Figure 10.** Validation of four differentially expressed genes (DEGs) by real-time reverse transcription-PCR (qRT-PCR). The expression patterns of four DEGs respectively in control and CMNV group were determined by qRT-PCR and compared with those obtained by RNA-seq. Values are the means of three replicates and shown as means ± standard deviation (N = 3). *P < 0.05, ***P < 0.001.

## Supplementary Tables

**Supplementary Table 1. Names and abbreviations for viral species of *Nodaviridae***

| **Virus** | **Abbreviation** | **GenBank No.** |
| --- | --- | --- |
| Covert mortality nodavirus | CMNV | KM112247 |
| Flock House virus | FHV | NP_689444 |
| Black beetle virus | BBV | YP_053043 |
| *Macrobrachium rosenbergii* nodavirus_China strain | MrNV China | AAQ54758 |
| *Macrobrachium rosenbergii* nodavirus_Australia strain | MrNV Australia | AEY63648 |
| *Macrobrachium rosenbergii* nodavirus_Malaysia strain | MrNV Malaysia | AEQ39078 |
| *Penaeus vannamei* nodavirus | PvNV | YP_004207810 |
| *Drosophila melanogaster* American nodavirus | DmANV | ACU32794 |
| Nodamura virus | NoV | NP_077730 |
| Boolarra virus | BoV | NP_689439 |
| Pariacoto virus | PaV | NP_620109 |
| Striped jack nervous necrosis virus | SJNNV | NP_599247 |
| Tiger puffer nervous necrosis virus | TPNNV | YP_003288759 |
| Atlantic halibut nodavirus | AHNV | AAY34458 |
| Golden pompano nervous necrosis virus | GPNNV | ACX54065 |
| Atlantic cod nodavirus | ACNV | ABR23192 |
| Japanese flounder nervous necrosis virus | JFNNV | ACN58225 |
| Dragon grouper nervous necrosis virus | DGNNV | AAU85148 |
| Barfin flounder nervous necrosis virus | BFNNV | YP_003288756 |
| Redspotted grouper nervous necrosis virus | RGNNV | ACX69744 |

Note: GenBank No. indicated the GenBank accession numbers of the amino acid sequence of RNA-dependent RNA polymerase used in this study.

**Supplementary Table 2. Statistics of the filtering raw data**

| **Sample** | **RawDatas** | **CleanData(%)** | **Adapter(%)** | **LowQuality(%)** | **polyA(%)** | **N(%)** |
| --- | --- | --- | --- | --- | --- | --- |
| T_CMNV_1 | 56950248 | 56729838 (99.61%) | 41404 (0.07%) | 179002 (0.31%) | 0 (0.00%) | 4 (0.00%) |
| T_CMNV_2 | 48588042 | 48424842 (99.66%) | 17634 (0.04%) | 145498 (0.30%) | 0 (0.00%) | 68 (0.00%) |
| T_CMNV_3 | 40202528 | 40054342 (99.63%) | 17884 (0.04%) | 130260 (0.32%) | 0 (0.00%) | 42 (0.00%) |
| CK-1 | 66889636 | 66757388 (99.80%) | 24012 (0.04%) | 108198 (0.16%) | 0 (0.00%) | 38 (0.00%) |
| CK-2 | 49473516 | 49337568 (99.73%) | 25146 (0.05%) | 110742 (0.22%) | 0 (0.00%) | 60 (0.00%) |
| CK-3 | 38453316 | 38355818 (99.75%) | 17966 (0.05%) | 79524 (0.21%) | 0 (0.00%) | 8 (0.00%) |

**Supplementary Table 3. Ribosomal comparing statistics of the sequenced reads**

| **Sample** | **Clean_reads** | **Mapped_Reads(%)** | **Unmapped_Reads(%)** |
| --- | --- | --- | --- |
| T_CMNV_1 | 56729838 | 1667946 ( 2.94% ) | 55061892 ( 97.06% ) |
| T_CMNV_2 | 48424842 | 1932956 ( 3.99% ) | 46491886 ( 96.01% ) |
| T_CMNV_3 | 40054342 | 1170330 ( 2.92% ) | 38884012 ( 97.08% ) |
| CK-1 | 66757388 | 1418234 ( 2.12% ) | 65339154 ( 97.88% ) |
| CK-2 | 49337568 | 2358640 ( 4.78% ) | 46978928 ( 95.22% ) |
| CK-3 | 38355818 | 1893208 ( 4.94% ) | 36462610 ( 95.06% ) |

**Supplementary Table 4. Statistics of the bases from the clean data**

| **Sample** | **RawData(bp)** | **BF_Q20(%)** | **BF_Q30(%)** | **BF_N(%)** | **BF_GC(%)** | **CleanData(bp)** | **AF_Q20(%)** | **AF_Q30(%)** | **AF_N(%)** | **AF_GC(%)** |
| --- | --- | --- | --- | --- | --- | --- | --- | --- | --- | --- |
| T_CMNV_1 | 8542537200 | 8327606013 (97.48%) | 7989086172 (93.52%) | 56922 (0.00%) | 4531185459 (53.04%) | 8461110300 | 8268152939 (97.72%) | 7938140254 (93.82%) | 56391 (0.00%) | 4485218376 (53.01%) |
| T_CMNV_2 | 7288206300 | 7106959541 (97.51%) | 6807869823 (93.41%) | 30653 (0.00%) | 3889445552 (53.37%) | 7229156029 | 7060068785 (97.66%) | 6766450669 (93.60%) | 29525 (0.00%) | 3855464866 (53.33%) |
| T_CMNV_3 | 6030379200 | 5872047343 (97.37%) | 5616602628 (93.14%) | 25335 (0.00%) | 3190554795 (52.91%) | 5983338733 | 5835743110 (97.53%) | 5584860160 (93.34%) | 24571 (0.00%) | 3163560102 (52.87%) |
| CK-1 | 10033445400 | 9819605508 (97.87%) | 9440367105 (94.09%) | 97054 (0.00%) | 5412908441 (53.95%) | 9960631005 | 9758515222 (97.97%) | 9385550479 (94.23%) | 95959 (0.00%) | 5372877801 (53.94%) |
| CK-2 | 7421027400 | 7231611802 (97.45%) | 6918641700 (93.23%) | 31124 (0.00%) | 3896150526 (52.50%) | 7385619544 | 7206582925 (97.58%) | 6897673711 (93.39%) | 30221 (0.00%) | 3875763452 (52.48%) |
| CK-3 | 5767997400 | 5648092526 (97.92%) | 5432587264 (94.18%) | 43532 (0.00%) | 3049545973 (52.87%) | 5715459557 | 5604667774 (98.06%) | 5393817110 (94.37%) | 43028 (0.00%) | 3019994842 (52.84%) |

**Supplementary Table 5. Sequences of the primers used in the experimental validation of DEGs**

| **Gene ID** | **Sequence（5’ to 3’）** | **Product size (bp)** |
| --- | --- | --- |
| 18s | TATACGCTAGTGGAGCTGGAA | 147 |
|  | GGGGAGGTAGTGACGAAAAAT |  |
| LOC113803353 | CCACCATGTACCCTGGTATT | 118 |
|  | CCGATCCAGACGGAGTATTT |  |
| LOC113806545 | CGGTAACTCGGAGCCAAATAA | 125 |
|  | CCACGCTCTTCTCACATACAG |  |
| LOC113808838 | GCAATGCTGGACAAGAGAATG | 110 |
|  | TCACCAAGGAGGTGGTAGTA |  |
| LOC113810077 | CCACCTTATTGGCTTCTCTCTC | 86 |
|  | CCAGACCTGTAATCCTGTCAAC |  |
